# Cardiometabolic function in retired night shift workers and retired day workers

**DOI:** 10.1038/s41598-022-20743-1

**Published:** 2023-03-30

**Authors:** Brian N. Chin, H. Matthew Lehrer, Eunjin Lee Tracy, Emma Barinas-Mitchell, Kristine A. Wilckens, Lucas W. Carroll, Daniel J. Buysse, Martica H. Hall

**Affiliations:** 1 Department of Psychiatry, University of Pittsburgh, Western Psychiatric Hospital, 3811 O’Hara Street, Pittsburgh, PA 15213 USA; 2 Department of Epidemiology, University of Pittsburgh, Pittsburgh, PA 15260 USA; 3 Department of Psychology, Trinity College, Hartford, CT 06106 USA

**Keywords:** Risk factors, Epidemiology

## Abstract

Night shift work is associated with poor cardiometabolic outcomes, even post-retirement. However, the characteristics of cardiometabolic function in retired night shift workers (RNSW) compared to retired day workers (RDW) are not well-understood. Rigorous characterization of cardiometabolic dysfunction in RNSW and RDW will inform targeted risk stratification for RNSW. This observational study evaluated whether RNSW (*n* = 71) had poorer cardiometabolic function than RDW (*n* = 83). We conducted a multimodal assessment of cardiometabolic function including metabolic syndrome prevalence, brachial artery flow-mediated dilation, and carotid intima-media thickness. Main analyses tested overall group differences. Sex-stratified follow-up analyses tested group differences separately in men and women. RNSW had 2.6-times higher odds of metabolic syndrome prevalence than RDW in unadjusted analyses (95% CI  [1.1,6.3]); this association was not significant when adjusting for age, race and education. RNSW and RDW (M_age_ = 68.4; 55% female) did not differ in percent flow-mediated dilation or carotid intima-media thickness. In sex-stratified analyses, women RNSW had 3.3-times higher odds of having high body mass index than women RDW (95% CI  [1.2,10.4]). Men RNSW had 3.9-times higher odds of having high triglycerides than men RDW (95% CI [1.1,14.2]). No other group differences were observed. We found mixed evidence that night shift work exposure was associated with cardiometabolic dysfunction in retirement, possibly in a sex-specific manner.

## Introduction

Nearly 20% of the world’s labor force routinely engages in work at night during typical sleeping hours (i.e. between midnight and 6:00 a.m.)^1,2^. Night shift work affects occupations across the socioeconomic spectrum including retail, health care, transportation, food service and protective service. At the heart of the public health challenge posed by night shift work is the fact that humans are a diurnal species, hard-wired to spend the night asleep and spend the day awake^3^. Night shift work represents a significant stress to sleep–wake systems and circadian rhythms because it necessitates sleep and wakefulness during times that are not conducive to either of these states, both in terms of internally regulated circadian mechanisms and environmental factors (e.g. light and noise). Moreover, both night and rotating shift workers often revert to a diurnal orientation during days off, and thus experience repeated circadian phase shifts on a weekly basis^4^ further impairing sleep–wake function^5^. These biological disturbances may explain findings of increased risk for adverse cardiometabolic health outcomes among night shift workers, including hypertension^6^, obesity^6,7^, diabetes^8,9^, metabolic syndrome^10^, and cardiovascular events and stroke^11^. Moreover, some studies have found that the adverse effects of night shift work on metabolic function differ by sex^12,13^. This underscores the importance of understanding sex differences in vulnerability to night shift work.

Retired night shift workers (RNSW) demonstrate higher metabolic syndrome prevalence compared to retired day workers (RDW) even when retirees are free to sleep at desired times^13,14^. Thus, long-term exposure to repeated phase shifts associated with night shift work may induce persistent impairments in cardiometabolic function even after return to a diurnal sleep–wake cycle. Increased understanding of how night shift work impacts cardiometabolic function in retirement could inform early detection of cardiovascular disease risk, as well as targeted intervention and rehabilitation strategies to improve cardiometabolic outcomes in millions of retired older adults.

This investigation evaluated whether RNSW had poorer cardiometabolic function than RDW. We conducted a multimodal assessment of cardiometabolic function that included metabolic syndrome prevalence (and prevalence of its subcomponents), brachial artery flow-mediated dilation (measure of endothelial function), and carotid intima-media thickness (measure of arterial remodeling). We hypothesized that RNSW (compared with RDW) would exhibit poorer cardiometabolic function (i.e. greater metabolic syndrome prevalence, less flow-mediated dilation and greater carotid intima-media thickness). Consistent with earlier evidence suggesting that the adverse effects of night shift work on cardiometabolic function may be more pronounced among women than among men^12,15^, we tested our hypothesis in the overall sample and then separately in men and in women.

## Methods

### Participants and procedures

Participants were older adults from the greater Pittsburgh area. Data collection for this study was conducted between April 2016 and March 2020. Retired men and women ages 60 and over were recruited via community research registries, print advertising (fliers, brochures, cultural playbills and magazine articles), television advertising and referrals. Study protocols were approved by the University of Pittsburgh Institution Review Board. All procedures were performed in accordance with relevant guidelines and regulations. Participants provided informed consent and received financial compensation up to 500 USD. Target sample size was determined by conducting a power analysis using pilot data from our study of 98 older adults (31.6% night shift work exposure). Power was calculated with a two-tailed regression model with three covariates and *α* = 0.05. Power analyses of the metabolic syndrome using an expected group difference of 23.3% estimated that 71 participants per group were needed for 80% power to detect a significant group difference using a logistic regression model. Power analyses of flow-mediated dilation using an expected group difference of 2.0% estimated that 77 participants per group were needed for 80% power to detect a significant group difference using a linear regression model.

Inclusion criteria were age ≥ 60, English speaking and writing, and being currently retired (defined as not working for pay > 10 h/week within the past year). Exclusion criteria were: severe cardiometabolic disease (e.g. myocardial infarction, insulin-dependent diabetes); severe obstructive sleep apnea; degenerative or other severe neurological diseases that could be exacerbated by sleep deprivation; history of stroke or transient ischemic attack; severe and/or untreated psychiatric disorder (e.g. bipolar disorder, schizophrenia, and untreated major depression); extremely advanced or delayed sleep hours (bedtime earlier than 8:00 P.M. or later than 4:00 A.M.; wake time earlier than 4:00 A.M. or later than noon); current use of beta blockers, insulin, antipsychotic drugs, steroids, anticoagulants, aspirin and/or anticonvulsants; regular use (> 3 doses/week) of hypnotics, melatonin, or other medications that affect sleep, non-steroidal anti-inflammatory drugs, opiates, and/or stimulants; consumption of > 14 alcohol drinks/week or > 3 caffeinated drinks (~ 300 mg) per day; and current smoking. Eligibility was assessed via a standardized telephone screening interview conducted by a trained staff member, two in-person laboratory visits that included a physical examination with a study physician, home sleep diaries, and one night of home sleep apnea screening with an ApneaLink Air device (ResMed, San Diego, CA)^16^ or laboratory-based overnight sleep study.

Participants deemed eligible after all screening procedures were scheduled for an in-person visit to the University of Pittsburgh Ultrasound Research Laboratory for an assessment of cardiometabolic function. This visit occurred in the morning and participants were instructed to arrive fasted. A laboratory technician completed ultrasound assessments of participants’ brachial artery flow-mediated dilation and carotid artery intima-media thickness, and measured participants’ resting systolic and diastolic blood pressure. A fasting blood draw assessed circulating glucose, triglyceride, and high-density lipoprotein (HDL) cholesterol levels. Efforts to reduce potential sources of bias included blinding of the outcome assessors to participants’ work history, using identical inclusion and exclusion criteria for each group (except for night shift work exposure), and attempting to match groups on demographic characteristics. Finally, participants completed computerized self-report questionnaires assessing demographic characteristics.

## Measures

### Night shift work exposure

During the initial telephone screening a trained staff member conducted a detailed assessment of participants’ work history. During the in-person screening visit, participants also completed questionnaires and a work history interview that were used to ascertain more specific information about their past jobs and to verify their status as a RNSW or RDW. RNSW were individuals who had ≥ 7 years of night shift work exposure (i.e. working between midnight and  6:00 A.M. on a permanent or rotating basis). This cutoff value was selected because past studies have shown that the deleterious effects of night shift work on sleep, circadian rhythms, and health may be attenuated among retired night shift workers with fewer years of exposure^14,17^. RDW were individuals who had < 1.5 years of full-time night shift work exposure, with their remaining work history comprising daytime or afternoon shifts. Responses to the work history interview were also used to determine lifetime exposure to night shift work and time since last exposure to night shift work.

### Metabolic syndrome prevalence

Metabolic syndrome prevalence was defined based on International Diabetes Federation criteria^18^ of high body mass index (BMI) (defined as > 30 kg/m^2^) and any two of the following four risk factors: (1) high blood pressure (systolic blood pressure ≥ 130 mmHg or diastolic blood pressure ≥ 85 mmHg) or current use of antihypertensive medication, (2) high fasting glucose (≥ 100 mg/dl) or current use of antihyperglycemic medication, (3) high triglycerides (≥ 150 mg/dl) or current use of antilipemic agents (e.g. fibrates), and (4) low HDL cholesterol (< 50 mg/dl for women or < 40 mg/dl for men). BMI was assessed during the physical examination by measuring participants’ height and weight. Systolic and diastolic blood pressure was assessed using an automatic blood pressure cuff during the Ultrasound Research Laboratory study visit. Glucose, triglycerides, and HDL cholesterol were assessed via fasting blood draw. Current medications were assessed via telephone interview and then corroborated via in-person interview. For the purposes of the current study, each medication was categorized by a study physician as antihypertensive, antihyperglycemic, antilipemic agents (e.g. fibrates), or other. Metabolic syndrome prevalence was only calculated if a participant had complete data for all five components. Metabolic syndrome prevalence data were available from 150 to 154 participants (97.5%); four participants were missing data for at least one component of the metabolic syndrome: blood pressure (*n* = 1), fasting glucose (*n* = 2), triglycerides (*n* = 2), and HDL cholesterol (*n* = 3).

### Brachial artery flow mediated dilation

Endothelial function was assessed by measuring active vessel relaxation (dilation) in response to a standardized shear stress challenge^19^. Technicians placed a blood pressure cuff on the participant’s right arm below the antecubital fossa and connected the participant to an electrocardiogram (EKG) monitor. After 10 min of supine rest, high resolution B-mode ultrasound was used to visualize a 1 cm longitudinal segment of the brachial artery above the antecubital fossa. Images of the brachial artery were captured continuously for 20 s (baseline). Next, the blood pressure cuff was inflated to 50 mmHg above the resting SBP for 5 min and then deflated. Images of the brachial artery were then captured continuously for 3 min after cuff deflation (post-deflation). Using the semi-automated edge-detection software Brachial Analyzer for Research (MIA, University of Iowa), brachial arterial diameter at baseline and post-deflation was measured on the R wave as the distance between the far and near arterial wall media-adventitia interfaces by a single trained reader for all study participants. We assessed the percent maximum change in brachial artery diameter post-deflation relative to baseline. Flow-mediated dilation data were available from 146 to 154 (94.8%) participants; five participants did not complete the scan procedure and three participants’ data were excluded because of poor scan quality.

### Carotid intima-media thickness

Trained and certified technicians at the University of Pittsburgh Ultrasound Research Laboratory used a high resolution ultrasound system to obtain bilateral digitized B-mode images in end diastole of the distal common carotid artery (1 cm proximal to the carotid bulb), carotid bifurcation (the point in which the near and far walls of the common carotid are no longer parallel, extending to the flow divider), and the first centimeter of the internal carotid artery (measured from the tip of the flow divider)^20,21^. Semiautomated reading software^22^ was used to generate mean intima-media thickness measures of the near and far walls of the common carotid artery and the far walls of the carotid bifurcation and internal carotid artery. Mean intima-media thickness at each location was averaged to calculate an overall measure of carotid intima-media thickness. Carotid intima-media thickness data were available from 151 to 154 participants (98.1%); two participants did not complete this procedure and one participant’s data were excluded because of poor scan quality.

## Data analysis

We conducted descriptive analyses of the sample by calculating mean (*M*) and standard deviation (*SD*) values in the full sample and stratified by group. We evaluated group differences in demographic characteristics using independent samples *t* tests for continuous variables and chi-squared tests for categorical variables.

We evaluated group differences (independent variable: RNSW vs. RDW) in cardiometabolic function (dependent variables: metabolic syndrome prevalence, flow-mediated dilation, and carotid intima-media thickness). Covariates were selected a priori because of their associations with night shift work exposure and cardiometabolic function in past studies^23^. Analyses were adjusted for self-reported age (continuous), race (categorical: non-Hispanic white—yes/no), and years of education (continuous). Logistic regression models were used to evaluate whether RNSW and RDW differed in prevalence of the metabolic syndrome and each of its components. Linear regression models were used to evaluate whether RNSW and RDW differed in flow-mediated dilation and carotid intima-media thickness. Primary analyses were performed across the entire sample. Follow up analyses stratified by sex were performed separately in women and men. We report both unadjusted analyses and analyses that are adjusted for a priori covariates.

## Results

154 of the 309 individuals who completed the screening procedures also completed the cardiometabolic assessment (49.8%). Characteristics of the final sample (*N* = 154) are shown in Table 1. The final sample consisted of 83 RDW (46 women, 37 men) and 71 RNSW (39 women, 32 men). RDW reported more years of education than RNSW, *t *(152) = 7.03, *p* < 0.001. RDW and RNSW did not differ in age, *t *(152) = 1.76, *p* = 0.08, sex, *χ*^2^ (1) = 0.00, *p* = 0.95, or race, *χ*^2^ (1) = 1.19, *p* = 0.28.

**Table 1:** Descriptive characteristics of the sample of retired night shift workers and retired day workers in the United States (2016–2019).

	Full sample (*N* = 154)	RNSW (*n* = 71)	RDW (*n* = 83)
Age, years	68.4 (5.4)	67.6 (5.0)	69.2 (5.8)
**Sex, % (*n*)**			
Women	55.2 (85)	54.9 (39)	55.4 (46)
Men	44.8 (69)	45.1 (32)	44.6 (37)
**Race, % (*n*)**			
White	86.4 (133)	83.1 (59)	89.2 (74)
Black	11.0 (17)	16.9 (12)	6.0 (5)
Other race	2.6 (4)	0 (0)	4.8 (4)
Education, years	15.8 (2.1)	14.7 (1.8)	16.8 (1.9)
Body mass index, kg/m^2^	27.7 (5.5)	27.4 (4.7)	28.0 (6.1)
Systolic blood pressure, mmHg	133.2 (15.6)	135.6 (17.6)	131.3 (13.5)
Diastolic blood pressure, mmHg	78.0 (9.6)	82.2 (9.8)	74.6 (8.1)
Glucose, mg/dl	97.6 (14.9)	102.1 (14.6)	94.0 (14.3)
Triglycerides, mg/dl	108.9 (49.9)	106.6 (53.0)	110.9 (47.5)
HDL-cholesterol, mg/dl	59.8 (16.5)	51.9 (13.2)	66.1 (16.2)
Metabolic syndrome, %	16.7 (25)	23.9 (16)	10.8 (9)
High body mass index, %	26.0 (40)	32.4 (23)	20.5 (17)
High blood pressure, %	61.4 (94)	60.0 (42)	62.7 (52)
High fasting glucose, %	42.1 (64)	49.3 (34)	36.1 (30)
High triglycerides, %	17.8 (27)	23.2 (16)	13.3 (11)
Low HDL-cholesterol, %	14.3 (22)	16.2 (11)	13.3 (11)
Flow-mediated dilation, % change	5.1 (2.7)	4.7 (2.6)	5.4 (2.8)
Carotid intima-media thickness, mm	0.8 (0.2)	0.8 (0.2)	0.8 (0.2)

Data are presented as *M *(SD) or % (*n*).

*RNSW* = retired night shift workers, *RDW* = retired day shift workers.

Mean night shift work exposure was 26.3 years (SD = 11.0 years) in RNSW and 0.2 years (SD = 0.6 years) in RDW. 31.0% of RNSW had 7–19 years of night shift work exposure; 69.0% of RNSW had 20 + years of night shift work exposure. Mean time since night shift work was 9.3 years (SD = 9.0 years) in RNSW.

Distributions and tests of normality for continuous cardiometabolic risk variables are shown in the online supplement. Cardiometabolic risk variables were normally distributed.

## Metabolic syndrome prevalence

Prevalence rates in the overall sample were 16.7% for metabolic syndrome, 26.0% for high body mass index, 61.4% for high blood pressure, 42.1% for high fasting glucose, 17.8% for high triglycerides, and 14.6% for low HDL cholesterol.

Table 2 summarizes the results of logistic regression models testing whether RNSW (compared with RDW) had greater prevalence of the metabolic syndrome and/or its individual components, including analyses stratified by sex. In unadjusted analyses of the full sample, RNSW were more likely than RDW to meet criteria for prevalent metabolic syndrome (OR 2.58, 95% CI 1.06, 6.29); however, this association was attenuated by inclusion of covariates for age, race, and education (OR 2.12, 95% CI 0.77, 5.81). RNSW and RDW did not differ in their likelihood of meeting criteria for any individual component of the metabolic syndrome.

**Table 2:** Results of logistic regression models comparing the likelihood of metabolic syndrome prevalence by group (retired night shift workers vs. retired day workers).

	Full sample (*N* = 154)	Men (*n* = 69)	Women (*n* = 85)
**Unadjusted**			
Metabolic syndrome^a^	**2.58 (1.06, 6.29)**	2.06 (0.52, 8.12)	3.04 (0.94, 9.87)
Body mass index	1.64 (0.77, 3.48)	0.87 (0.28, 2.68)	**3.30 (1.22, 8.93)**
Blood pressure^b^	0.89 (0.47, 1.72)	0.70 (0.26, 1.88)	1.08 (0.45, 2.59)
Glucose^c^	1.72 (0.89, 3.34)	1.72 (0.65, 4.58)	1.86 (0.72, 4.78)
Triglycerides^c^	1.98 (0.85, 4.60)	**3.93 (1.09, 14.16)**	1.05 (0.32, 3.42)
HDL-cholesterol^d^	1.26 (0.51, 3.12)	0.57 (0.13, 2.52)	2.19 (0.65, 7.35)
**Age-, race-, and education-adjusted**			
Metabolic syndrome^a^	2.12 (0.77, 5.81)	2.10 (0.38, 11.50)	2.23 (0.60, 8.25)
Body mass index	1.65 (0.70, 3.89)	0.77 (0.19, 3.16)	3.02 (0.95, 9.59)
Blood pressure^b^	0.77 (0.36, 1.64)	0.62 (0.18, 2.16)	0.89 (0.33, 2.40)
Glucose^c^	1.58 (0.75, 3.34)	1.69 (0.51, 5.67)	1.65 (0.57, 4.78)
Triglycerides^c^	1.58 (0.60, 4.19)	1.68 (0.33, 8.64)	1.27 (0.33, 4.94)
HDL-cholesterol^d^	0.97 (0.34, 2.72)	0.17 (0.02, 1.41)	1.29 (0.32, 5.09)

Results are presented as odds ratios and 95% confidence intervals comparing retired night shift workers (RNSW) to retired day workers (RDW).

Bolded numbers indicate statistical significance.

^a^Complete metabolic syndrome data were available from 150 participants.

^b^Blood pressure data were available from 153 participants.

^c^Glucose and triglyceride data were available from 152 participants.

^d^HDL-cholesterol data were available from 151 participants.

In sex-stratified analyses, we did not find group differences in metabolic syndrome prevalence in RNSW compared with RDW among men (OR 2.06, 95% CI 0.52, 8.12) or among women (OR 3.04, 95% CI 0.94, 9.87). Follow-up analyses that separately examined each component of the metabolic syndrome showed that women RNSW were 3.30-times more likely (95% CI 1.22, 8.93) than women RDW to meet criteria for high body mass index; however, this association was attenuated by inclusion of covariates for age, race and education (OR 3.02, 95% CI 0.95, 9.59). However, women RNSW and women RDW did not differ in likelihood of meeting criteria for other metabolic syndrome components. In unadjusted analyses, men RNSW were 3.9-times more likely (95% CI 1.09, 14.16) than men RDW to meet criteria for high triglycerides; however, this association was attenuated by inclusion of covariates for age, race, and education (OR 1.68, 95% CI 0.33, 8.64). Men RNSW and men RDW did not differ in likelihood of meeting criteria for other metabolic syndrome components.

The online supplement shows the results of secondary linear regression analyses testing group differences in continuous metabolic syndrome outcome variables. We did not find evidence for group differences in continuous metabolic syndrome outcome variables in the unadjusted or covariate-adjusted linear regression analyses.

## Brachial artery flow-mediated dilation

Table 3 summarizes the results of linear regression models testing whether RNSW (vs. RDW) had poorer endothelial function as indicated by (smaller) percent flow-mediated dilation, including analyses stratified by sex. In the full sample, RNSW and RDW did not differ in their percent dilation (*B* = − 0.72, 95% CI − 1.75, 0.30; RNSW: *M* = 4.7, *SD* = 2.6, RDW: *M* = 4.7, *SD* = 2.8).

**Table 3:** Results of linear regression models testing associations of group (retired night shift workers vs. retired day workers) with flow-mediated dilation and carotid intima-media thickness.

	Full sample (*N* = 154)	Men (*n* = 69)	Women (*n* = 85)
**Unadjusted**			
Flow-mediated dilation^a^ (% change)	− 0.64 (− 1.53, 0.25)	− 0.92 (− 2.18, 0.34)	− 0.32 (− 1.57, 0.93)
Carotid intima-media thickness^b^	− 0.00 (− 0.06, 0.06)	− 0.03 (− 0.13, 0.07)	0.02 (−0.04, 0.09)
**Age-, race-, and education-adjusted**			
Flow-mediated dilation^a^ (% change)	− 0.72 (− 1.75, 0.30)	− 1.51 (− 3.06, 0.05)	− 0.43 (− 1.87, 1.00)
Carotid intima-media thickness^b^	0.00 (− 0.06, 0.07)	0.04 (− 0.08, 0.15)	− 0.01 (− 0.08, 0.06)

Results are presented as unstandardized regression coefficients and 95% confidence intervals comparing retired night shift workers (RNSW) to retired day workers (RDW).

^a^Flow-mediated dilation data were available from 146 participants.

^b^Carotid intima-media thickness data were available from 151 participants.

In sex-stratified analyses, men RNSW and men RDW did not differ in their percent dilation (*B* = − 1.51, 95% CI − 3.06, 0.05; RNSW: *M* = 5.0, *SD* = 2.9, RDW: *M* = 4.1, *SD* = 2.1). Women RNSW and women RDW did not differ in their percent dilation (*B* = − 0.43, 95% CI − 1.87, 1.00; RNSW: *M* = 5.3, *SD* = 2.9, RDW: *M* = 5.6, *SD* = 2.7).

## Carotid intima-media thickness

Table 3 summarizes the results of linear regression models testing whether RNSW (vs. RDW) had greater intima-media thickness, including analyses stratified by sex. Carotid intima-media thickness did not differ between RNSW and RDW in the full sample (*B* = − 0.00, 95% CI − 0.06, 0.06; RNSW: *M* = 0.8 mm, *SD* = 0.2, RDW: *M* = 0.8 mm, *SD* = 0.2) or in sex-stratified analyses (men: *B* = − 0.03, 95% CI − 0.13, 0.07; women: *B* = 0.02, 95% CI − 0.04, 0.09).

## Discussion

This observational study evaluated whether retired night shift workers (RNSW) and retired day workers (RDW) differed in their cardiometabolic function. In the full sample, RNSW and RDW did not differ in their metabolic syndrome prevalence, flow-mediated dilation, or carotid intima-media thickness after controlling for age, race, and education. In sex-stratified analyses, we found that women RNSW had greater prevalence of high body mass index than women RDW, and that men RNSW had greater prevalence of high triglycerides than men RDW. In contrast, men RNSW and men RDW did not differ in prevalence of high body mass index, and women RNSW and women RDW did not differ in prevalence of high triglycerides. No group differences for metabolic syndrome prevalence, flow-mediated dilation, or carotid intima-media thickness were observed in women or in men.

This study evaluated whether RNSW had greater prevalence of the metabolic syndrome and its components compared with RDW. In unadjusted analyses, RNSW had 2.6-times higher odds of metabolic syndrome prevalence than RDW; however, this association was not statistically significant after inclusion of covariates for age, race, and education. It is likely that the covariate-adjusted logistic regression models were underpowered to detect an association. Consistent with this explanation, RNSW had 2.1-times higher odds of metabolic syndrome prevalence than RDW in the covariate-adjusted analyses. Results of the unadjusted analyses are consistent with earlier studies which found that RNSW demonstrate higher metabolic syndrome prevalence compared to RDW^13,14^. However, the lack of significant differences in metabolic syndrome prevalence among men is contrary to an earlier study which found that men night shift workers had greater metabolic syndrome prevalence than men day workers^13^. Future research is needed to elucidate the mechanisms linking night shift work and metabolic syndrome prevalence, and to understand mechanisms of sex differences in this association. For example, circadian misalignment (i.e., between environmental, behavioral, and biological processes) has been shown to result in glucose dysmetabolism^24^ and may represent one mechanism linking night shift work exposure and cardiometabolic dysfunction^25–27^. Engagement in unhealthy lifestyle behaviors (i.e. low physical activity, poor diet, and smoking) is another hypothesized mechanism accounting for this association^27^.

To our knowledge, this study was the first to evaluate whether RNSW have poorer endothelial function and greater carotid intima-media thickness than RDW. We found that RNSW and RDW did not differ in their endothelial function or in their carotid intima-media thickness. Our findings may suggest that night shift work is not strongly associated with changes in endothelial function or arterial structure. These results contrast with those of a recent study of 3582 Chinese steelworkers which found that current night shift workers had greater carotid intima-media thickness than current day workers^28^. One possible explanation for this difference is that our study’s smaller sample may have been underpowered to detect a small-to-moderate difference in cardiometabolic function between RNSW and RDW.

## Limitations and future directions

This study’s strengths include objective measures of clinical and subclinical cardiometabolic function and investigation of past shift work exposure in two distinct groups of retirees. However, the current results should be interpreted in the context of several limitations. First, this study was cross-sectional, and it is unknown whether any observed differences between RNSW and RDW represent a *persistent* effect of night shift work. Differences could emerge during night shift work exposure or during retirement; prospective studies are ideal to probe this question. Moreover, it is possible that observed differences are driven by different individuals self-selecting into night shift work (vs. day work), although eligibility criteria for our study excluded individuals with medical (e.g. sleep apnea) or behavioral (e.g. smoking, substance abuse) characteristics that might explain differences in cardiometabolic function in RNSW and RDW. Second, our study’s sample consisted of relatively healthy retired adults, and thus we may have underestimated the impact of night shift work exposure on cardiometabolic function because of survivorship bias and selection bias. RNSW in our study may have consisted of retirees who were healthier and more resilient to circadian misalignment than typical night shift workers. Consistent with this possibility, the prevalence of metabolic syndrome in our sample (16.7%) was lower than the prevalence in the general population of the United States (36.9% in 2015–2016)^29^. Another explanation for these null effects is that the impact of night shift work exposure on cardiometabolic function may decrease with longer times since cessation of night shift work. Consistent with this explanation, mean time since night shift work exposure in our study was nearly 10 years. Third, our study’s measure of brachial artery flow-mediated dilation did not account for individual shear rate which would have improved our ability to assess endothelial function. Fourth, our study defined the metabolic syndrome using body mass index instead of the more common measure of waist circumference.

## Conclusion

This study found mixed evidence that past night shift work exposure was associated with poorer cardiometabolic function. RNSW had 2.6-times higher odds of metabolic syndrome prevalence than RDW in unadjusted analyses; this association was not significant when adjusting for age, race and education. We also found that women RNSW had higher prevalence of high body mass index than women RDW, and that men RNSW had higher prevalence of high triglycerides than men RDW. These findings add to growing evidence that night shift work exposure is associated with cardiometabolic dysfunction even in retirement, and that such links may be influenced by biological sex. Future studies will determine if night shift work has persistent effects on cardiometabolic function, if these effects are moderated by sex, and if sex-specific intervention and rehabilitation strategies improve cardiometabolic function in men and women RNSW.

## Supplementary Information

Supplementary Information.

## Data Availability

Data from the current study are available from the corresponding author on reasonable request.
