# Supplementary material for: Cardiometabolic function in retired night shift workers and retired day workers
Source: Sci Rep. 2023 Mar 30;13:5204. doi: 10.1038/s41598-022-20743-1 (PMC10063655; doi:10.1038/s41598-022-20743-1)
Supplement: Supplementary file 1 — Supplementary Information. [file 41598_2022_20743_MOESM1_ESM.docx]

**Online Supplement to**

**“Cardiometabolic function in retired night shift workers and retired day workers”**

**A. Distributions and normality tests for continuous cardiometabolic risk outcomes**

***Body mass index***

Body mass index values were normally distributed with skewness = .91 and kurtosis = .58.


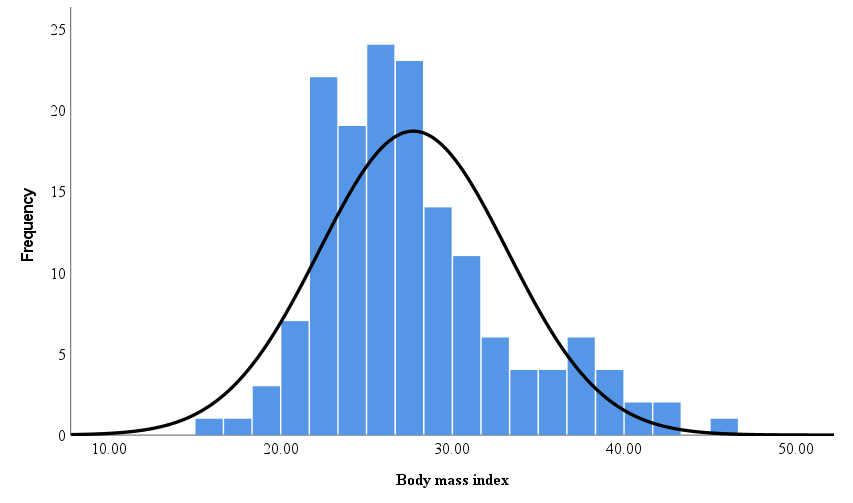


***Systolic blood pressure***

Systolic blood pressure values were normally distributed with skewness = .39 and kurtosis = .10.


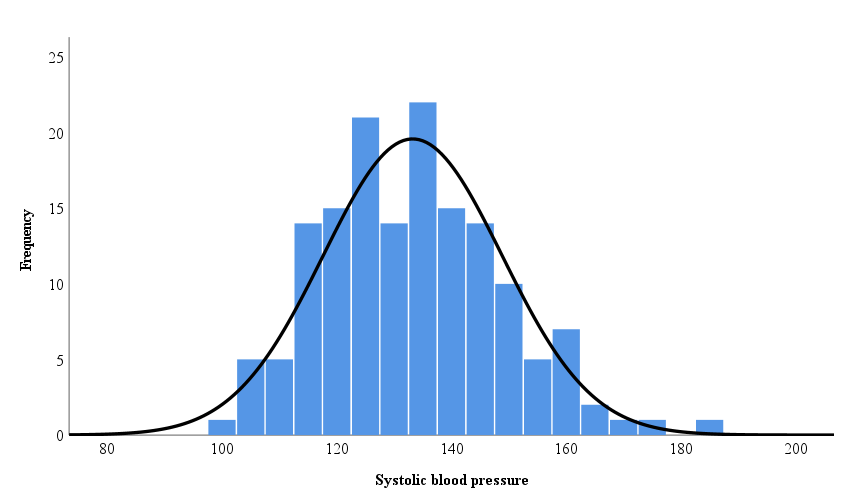


***Diastolic blood pressure***

Diastolic blood pressure values were normally distributed with skewness = .33 and kurtosis = .01.


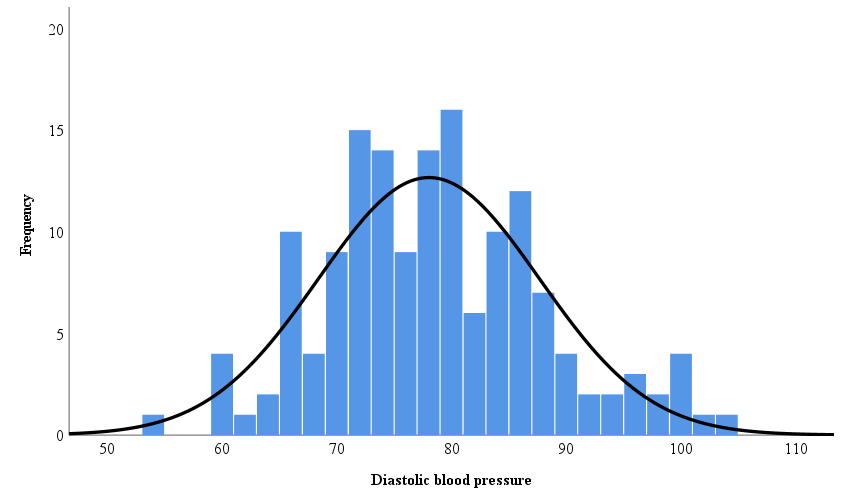


***Triglycerides***

Triglyceride values were normally distributed with skewness = 1.47 and kurtosis = 2.76.


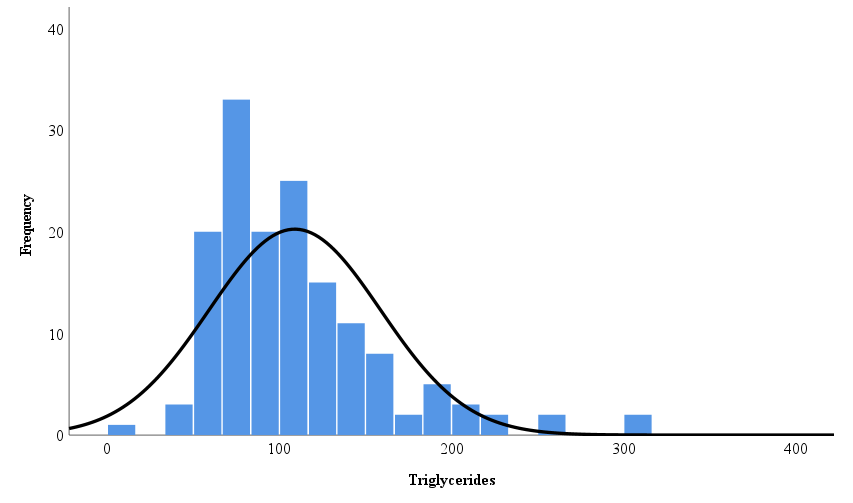


***Glucose***

Glucose values were relatively normally distributed with skewness = .69 and kurtosis = 3.43.


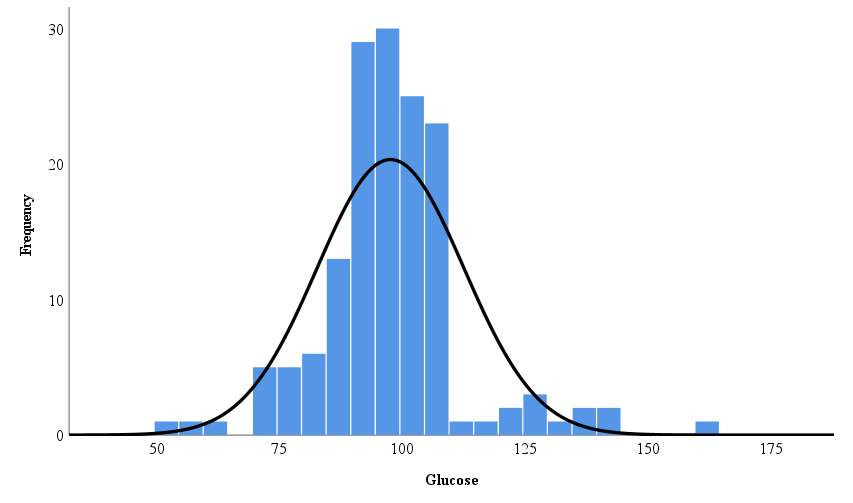


***HDL-cholesterol***

HDL-cholesterol values were normally distributed with skewness = .58 and kurtosis = .14.


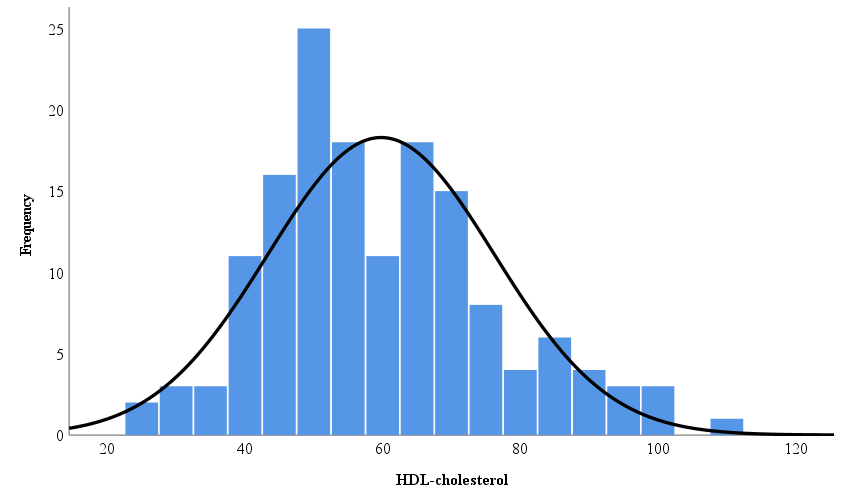


***Flow-mediated dilation***

Flow-mediated dilation values were normally distributed with skewness = .74 and kurtosis = .26.


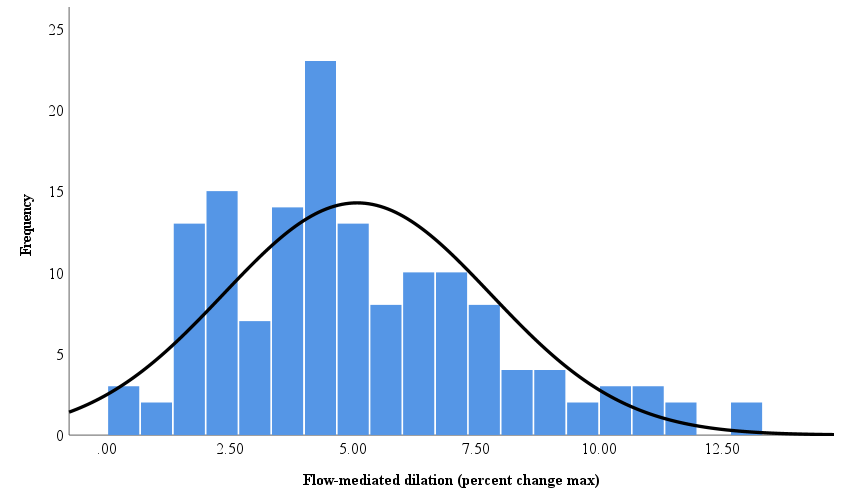


***Carotid intima-media thickness***

Carotid intima-media thickness values were normally distributed with skewness = 1.58 and kurtosis = 2.85.


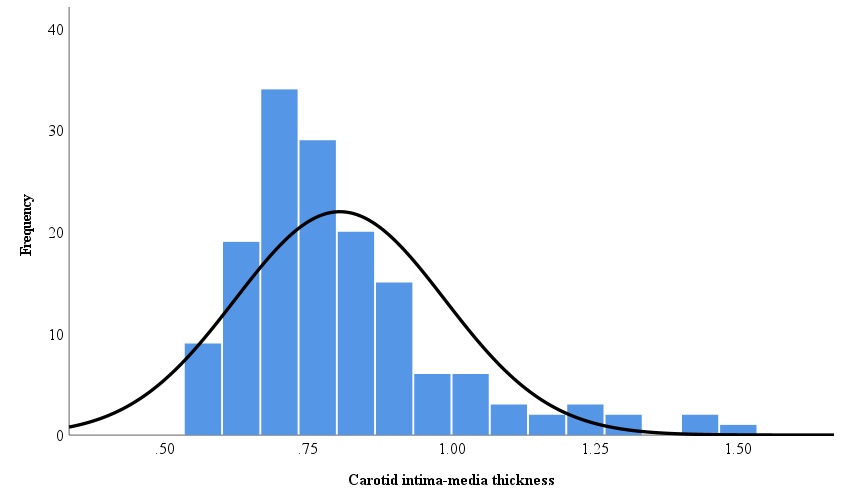


**B. Linear regressions testing the association of night shift work exposure and continuous values of the metabolic syndrome components**

Table S1

Unadjusted and covariate-adjusted associations of group (retired night shift workers vs retired day workers) with continuous values of the metabolic syndrome components

|  | Full sample  (*N* = 154) | Men  (*n* = 69) | Women  (*n* = 85) |
| --- | --- | --- | --- |
| **Unadjusted** |  |  |  |
| Body mass index | 1.14 (-0.60, 2.89) | -0.37 (-2.63, 1.88) | 2.38 (-0.22, 4.98) |
| Systolic blood pressure | -3.50 (-8.48, 1.48) | -8.01 (-16.31, 0.28) | 0.13 (-5.82, 6.07) |
| Diastolic blood pressure | -0.14 (-3.24, 2.96) | 1.58 (-3.15, 6.30) | -1.71 (-5.24, 1.82) |
| Triglycerides | 12.04 (-3.96, 28.04) | 27.31 (2.25, 52.38) | -0.32 (-21.16, 20.53) |
| Glucose | -1.35 (-6.16, 3.46) | 2.56 (-4.55, 9.67) | -4.57 (-10.75, 1.61) |
| HDL-cholesterol | -0.45 (-5.79, 4.89) | 1.46 (-5.07, 7.98) | -2.08 (-9.15, 4.99) |
|  |  |  |  |
| **Age-, race-, and education-adjusted** |  |  |  |
| Body mass index | 0.14 (-1.82, 2.09) | -0.95 (-3.76, 1.87) | 0.80 (-1.96, 3.57) |
| Systolic blood pressure | -2.62 (-8.29, 3.06) | -6.04 (-16.48, 4.41) | 0.27 (-6.38, 6.92) |
| Diastolic blood pressure | -0.80 (-4.40, 2.80) | 0.65 (-5.38, 6.67) | -1.53 (-5.53, 2.47) |
| Triglycerides | 5.84 (-12.24, 23.93) | 12.52 (-18.05, 43.09) | -1.79 (-25.39, 21.81) |
| Glucose | -2.64 (-8.21, 2.93) | 1.58 (-7.25, 10.41) | -6.16 (-13.11, 0.79) |
| HDL-cholesterol | 0.69 (-5.47, 6.85) | 2.36 (-5.80, 10.51) | 0.41 (-7.61, 8.43) |

Note. Results are presented as unstandardized regression coefficients and 95% confidence intervals comparing retired night shift workers to retired day workers. Data were available from 154 participants for body mass index, 153 participants for systolic and diastolic blood pressure, 152 participants for triglyceride and glucose, and 151 participants for HDL-cholesterol.
